# Fenofibrate in ophthalmology: therapeutic efficacy and mechanisms

**DOI:** 10.3389/jpps.2026.16995

**Published:** 2026-09-03

**Authors:** Sean Chung-Hei Ho, Isabelle Xin Yu Lee, Chang Liu, Mingyi Yu, Hong Chang Tan, Gavin Siew Wei Tan, Yu-Chi Liu

**Affiliations:** 1 University of Exeter Medical School, Exeter, United Kingdom; 2 Regenerative Therapy Group, Singapore Eye Research Institute, Singapore, Singapore; 3 Department of Endocrinology, Singapore General Hospital, Singapore, Singapore; 4 Surgical Retina Department, Singapore National Eye Centre, Singapore, Singapore; 5 Department of Cornea, Singapore National Eye Centre, Singapore, Singapore

**Keywords:** diabetic corneal neuropathy, diabetic retinopathy, fenofibrate, ophthalmology, peroxisome proliferator-activated receptor alpha

## Abstract

Ophthalmic diseases are a growing global cause of visual impairment, driven by population aging and the rising prevalence of diabetes mellitus. Fenofibrate, a peroxisome proliferator-activated receptor alpha (PPARα) agonist widely used for dyslipidemia, has emerged as a candidate for therapeutic repurposing in ophthalmology. This narrative review synthesizes current preclinical and clinical evidence on fenofibrate across anterior and posterior segment diseases, including diabetic corneal neuropathy, diabetic keratopathy, dry eye disease, corneal burns, Fuchs endothelial corneal dystrophy, diabetic retinopathy, diabetic macular edema, neovascular age-related macular degeneration, and central retinal artery occlusion. Across ocular disease models, fenofibrate demonstrates neuroprotective, vascular stabilizing, and tissue-remodeling effects. These actions appear to involve modulation of shared pathogenic pathways, including PPARα-dependentregulation of inflammatory signaling, mitochondrial and lipid metabolism, and preservation of epithelial, endothelial, neuronal, and vascular integrity. Clinical evidence is strongest for diabetic retinopathy, where randomized trials support a potential adjunctive role in slowing progression and reducing interventions in patients with early-to-moderate disease. Preliminary clinical evidence for diabetic corneal neuropathy is promising but limited, whereas data for dry eye disease, corneal burns, Fuchs endothelial corneal dystrophy, neovascular age-related macular degeneration, and retinal ischemic injury remains preclinical. Emerging experimental ocular delivery strategies may improve local bioavailability while reducing systemic exposure. Overall, fenofibrate represents a biologically plausible adjunctive ophthalmic therapy, but further randomized trials, mechanistic studies, pharmacokinetic evaluation, and long-term safety assessments are required to define its therapeutic role beyond diabetic retinopathy.

## Introduction

Ophthalmic diseases are a major and growing global cause of visual impairment and blindness, and this burden is increasing alongside population aging and the rising prevalence of diabetes mellitus (DM) [1, 2]. Diabetic retinopathy (DR) remains a leading cause of vision loss in the working-age population and is projected to affect approximately 161 million individuals worldwide by 2045 [2, 3]. In parallel, age-related macular degeneration (AMD) is a leading cause of blindness among older adults [4], with the global burden expected to rise to nearly 288 million affected individuals by 2040 [5]. Corneal diseases, including diabetic corneal neuropathy (DCN), dry eye disease (DED), corneal burns and neovascularization (NV), and Fuchs endothelial corneal dystrophy (FECD), also contribute substantially to ocular morbidity, with DED causing chronic ocular discomfort and reduced quality of life, and endothelial and angiogenic corneal disorders contributing to visual impairment and blindness in severe cases [6–9]. Many of these disorders are progressive and ultimately compromise corneal transparency, retinal integrity, or visual function [10–12].

Despite advances in ophthalmic therapeutics, current treatment strategies remain limited in several important respects. Retinal laser photocoagulation, although effective in selected settings, is invasive and associated with patient discomfort and retinal scarring [13, 14]. Similarly, intravitreal anti-vascular endothelial growth factor (VEGF) therapy remains costly, invasive, and treatment-intensive, requiring repeated injections, while persistent edema and incomplete therapeutic response remain common in diabetic macular edema (DME) [15, 16]. In parallel, management of many corneal diseases remains largely supportive or symptomatic, often failing to address the underlying disease mechanism or prevent progression [17–20]. These limitations have increased interest in therapeutic repurposing strategies and the development of less invasive, disease-modifying approaches capable of targeting shared pathogenic pathways across ocular diseases.

## Peroxisome proliferator-activated receptors and fenofibrate

Peroxisome proliferator-activated receptors (PPARs) are ligand-activated nuclear transcription factors that regulate lipid metabolism, glucose homeostasis, inflammation, and oxidative stress across three major isoforms: PPARα, PPARγ, and PPARβ/δ [21, 22].

Among these, PPARα has attracted increasing interest in ophthalmology because of its regulatory effects on mitochondrial homeostasis, vascular permeability, angiogenesis, and lipid metabolism within ocular tissues [23–26]. Experimental studies have demonstrated altered ocular PPARα expression in diabetic corneas and retinas, while genetic or pharmacologic modulation of PPARα influences corneal nerve integrity, retinal neuroinflammation, vascular leakage, leukostasis, and ischemia-associated retinal injury [27–31]. Mechanistically, PPARα activation has been associated with suppression of pro-inflammatory signaling pathways including nuclear factor kappa-light-chain-enhancer of activated B cells (NF-κB) and cyclic GMP–AMP synthase–stimulator of interferon genes (cGAS–STING) signaling, within ocular tissues [28, 32, 33].

Fenofibrate is a synthetic PPARα agonist that is hydrolyzed *in vivo* to fenofibric acid (FA), its active metabolite [34]. Although fenofibrate is currently approved for the treatment of primary hypercholesterolemia and mixed dyslipidemia [34], increasing evidence suggests that its biological effects extend beyond systemic lipid lowering. In addition to improving serum lipid profiles, fenofibrate has demonstrated anti-inflammatory, antioxidant, anti-angiogenic, neuroprotective, anti-fibrotic, and barrier-stabilizing properties across multiple ocular disease models [35–39]. Given its non-invasive nature and well-characterized systemic safety profile in routine practice and clinical studies, it has emerged as an attractive candidate for therapeutic repurposing in ophthalmology [40, 41].

Interest in fenofibrate as an ophthalmic therapy was initially driven by findings from the Fenofibrate Intervention and Event Lowering in Diabetes (FIELD) and the Action to Control Cardiovascular Risk in Diabetes (ACCORD) studies [42–44]. Although FIELD was designed to assess cardiovascular outcomes, secondary ophthalmic analyses showed that, in patients with pre-existing retinopathy, fenofibrate significantly reduced the need for first retinal laser treatment when analysed independently, and reduced 2-step Early Treatment Diabetic Retinopathy Study (ETDRS) progression when analysed in a composite with new macular edema or retinal laser treatment [44]. Both effects appeared disproportionate to changes in plasma lipids [45]. Subsequent findings from ACCORD Eye further demonstrated that fenofibrate in combination with simvastatin, reduced progression of DR, both ETDRS-defined worsening and a composite including retinal laser and vitrectomy, in patients with pre-existing disease [42]. More recently, the LENS trial provided dedicated randomized evidence supporting fenofibrate in reducing DR and maculopathy progression [46], reinforcing growing interest in fenofibrate as a potential disease-modifying ophthalmic therapy.

Despite growing interest in fenofibrate in ophthalmology, the current literature remains fragmented across disease-specific studies, mechanistic investigations, and emerging drug-delivery approaches [37, 47–51]. Existing reviews have largely focused on DR [52–54], whereas newer evidence now spans corneal neuropathic, inflammatory, angiogenic, and endothelial disorders in addition to retinal disease [48, 50, 55, 56]. Importantly, the recent publication of the randomized controlled LENS trial has further expanded the clinical evidence base for fenofibrate in DR and highlights the need for an updated synthesis of the evolving literature [46]. Furthermore, the strength of evidence differs substantially between indications, with randomized clinical trial data available for DR, emerging early clinical evidence for DCN, and predominantly preclinical evidence for several other ophthalmic diseases [32, 35, 43, 46, 48, 57]. A consolidated cross-ophthalmology synthesis is therefore needed to distinguish clinically supported indications from experimental applications, identify shared mechanisms, and clarify the translational potential and limitations of fenofibrate in ophthalmology.

This narrative review synthesizes current preclinical and clinical evidence regarding fenofibrate across corneal and retinal ophthalmic diseases, with particular emphasis on mechanisms of action, strength of evidence by indication, translational relevance, and emerging ocular drug-delivery strategies. In addition, this review discusses current research gaps, safety considerations, and future therapeutic directions for fenofibrate in ophthalmology. To our knowledge, this is among the first narrative review to integrate fenofibrate evidence across both anterior and posterior segment diseases within a unified mechanistic and translational framework.

## Corneal anatomy and physiology

The cornea is the transparent anterior structure of the eye and contributes to roughly two-thirds of its refractive power by focusing light onto the retina [58]. The corneal epithelium serves as a protective barrier, facilitates oxygen and nutrient exchange, and maintains ocular surface integrity through its regenerative capacity [58]. Disruption of the corneal epithelium increases susceptibility to infection and may compromise vision [58]. Posterior to the epithelium lies the stroma proper, which constitutes approximately 90% of corneal thickness [59]. The stromal extracellular matrix is essential for maintaining corneal transparency, and its disruption during fibrosis, inflammation, or NV can result in stromal opacity and visual impairment [59, 60]. The corneal endothelium is the innermost layer of the cornea and maintains corneal hydration and transparency through ion transport-mediated fluid regulation [58, 61]. Unlike the corneal epithelium, the human corneal endothelium has very limited regenerative capacity *in vivo*, and endothelial cell density declines with age [58, 62]. Endothelial dysfunction results in progressive endothelial cell loss and impaired corneal deturgescence (the maintenance of relative dehydration essential for transparency) [63, 64].

Under normal conditions, the cornea is avascular and derives oxygen and nutrients primarily through diffusion from the tear film and atmosphere anteriorly and the aqueous humour posteriorly [58]. Maintenance of corneal avascularity is essential for transparency, as pathologic corneal NV can cause opacity and increased light scattering [65]. The cornea is densely innervated by sensory nerves that play essential neurotrophic roles in maintaining epithelial integrity, ocular surface homeostasis, and wound healing [66–68]. Corneal nerves release neuromodulators, including substance P (SP) and nerve growth factor (NGF), which promote epithelial repair and support ocular surface homeostasis [67, 68]. In DCN, reduced corneal innervation impairs epithelial wound healing and contributes to the development of keratopathy [10].

## Retinal anatomy and physiology

Posterior to the cornea lies the retina, a multilayered neurosensory tissue responsible for phototransduction and the initial processing of visual information [69]. The retina contains photoreceptors (rods and cones), interneurons (bipolar, horizontal, and amacrine cells), and retinal ganglion cells organized into ten distinct layers [69]. Rods mediate low-light (scotopic) vision and are concentrated in the peripheral retina, whereas cones mediate high-acuity (photopic) and color vision and are concentrated in the macula, the central region responsible for fine visual discrimination [69]. At the center of the macula lies the fovea, a pit containing exclusively densely packed cones that provides the highest visual acuity [69]. The retina is supported by a dual blood supply: the choroidal circulation nourishes the outer retina (photoreceptors and retinal pigment epithelium), while the central retinal artery supplies the inner retinal layers via superficial and deep capillary plexuses [70]. The inner blood-retinal barrier is formed by tight junctions of the retinal capillary endothelium, whereas the outer blood-retinal barrier is formed by tight junctions of the retinal pigment epithelium (RPE), which contributes to photoreceptor homeostasis and phagocytoses shed outer segments [71]. Retinal diseases such as DR and AMD are driven by interconnected inflammatory, oxidative, vascular, and neurodegenerative mechanisms [72].

## Search strategy and selection criteria

This structured narrative review was informed by a literature search conducted across PubMed, Cochrane Library, and Embase databases on 21 March 2026 to identify studies examining the effects of fenofibrate on corneal and retinal diseases. The search strategy employed relevant keywords and MeSH terms, including *fenofibrate, fenofibric acid, PPAR, PPARα, cornea, corneal diseases, diabetic corneal neuropathy, diabetic keratopathy, corneal injury, corneal endothelium, ocular surface, retina, retinopathy, retinal diseases, diabetic retinopathy, retinal injury, retinal vessels, dry eye syndromes, macular degeneration, and corneal dystrophy.*

The initial search identified 282 articles. Titles and abstracts were screened for relevance, and excluded if they were duplicates, did not involve fenofibrate or FA, did not mainly involve corneal or retinal diseases, or were review articles. To emphasize contemporary evidence, studies published before 2010 were also excluded. An exception was made for the FIELD trial due to its foundational role in establishing fenofibrate outcomes in DR. After screening, 47 articles were retained for detailed review. An additional 10 relevant studies were identified through manual screening of reference lists, resulting in a total of 57 articles informing the final narrative synthesis.

The structured search and screening process was used specifically to identify studies included in the narrative synthesis. Other publications, including review articles and earlier studies, were consulted where relevant to provide background information and contextual discussion, but were not part of the screened study set.

## Diabetic corneas and diabetic corneal neuropathy

DCN is a common complication of DM characterized by progressive corneal nerve degeneration, impaired epithelial repair, reduced corneal sensitivity, and ocular surface dysfunction [10]. Current management remains largely supportive and symptomatic, including lubricants and prophylactic antibiotics, without directly addressing the underlying neurodegenerative and inflammatory mechanisms driving disease progression [18]. Preclinical studies have shown that fenofibrate improves neural dysfunction and reduces neurovascular injury in models of diabetic neuropathy, suggesting potential therapeutic relevance for diabetic neuropathic complications [73]. Similar to peripheral nerves, corneal nerves are highly vulnerable to diabetic injury, and their degeneration disrupts sensory and trophic support required for maintaining epithelial integrity and ocular surface homeostasis [10]. Emerging preclinical and early clinical evidence further suggests that PPARα agonism may promote corneal nerve regeneration, enhance epithelial repair, preserve mitochondrial function, and suppress ocular surface inflammation in DCN [25, 27, 35, 48, 74, 75].

Mitochondrial dysfunction appears to play a central role in diabetes-associated impairment of corneal wound healing, as diabetic corneal epithelial cells exhibit reduced oxidative phosphorylation, impaired adenosine triphosphate (ATP) production, and decreased expression of mitochondrial markers [25]. In type 1 and type 2 diabetic mouse models, epithelial PPARα activation by fenofibrate improved mitochondrial respiration and restored markers of mitochondrial integrity in the cornea, including translocase of outer mitochondrial membrane 20, changes that were associated with enhanced corneal epithelial repair [25]. These findings suggest that fenofibrate may support the high metabolic demands required for epithelial proliferation and migration during corneal wound healing.

In parallel, fenofibrate has shown neuroprotective effects on corneal nerves in both preclinical models and early clinical studies [27, 48, 74]. Oral fenofibrate was associated with increased corneal nerve fiber density and attenuation of diabetes-related corneal nerve loss in diabetic animals and patients with diabetes [27, 48, 74]. Conversely, *Ppara* knockout mice demonstrated reduced corneal nerve fiber density, impaired corneal sensitivity, and increased corneal lesions, supporting an important role for corneal PPARα in maintaining corneal nerve homeostasis [27]. In a clinical study, 30 days of oral fenofibrate treatment significantly improved corneal nerve fiber density, corneal nerve fiber width, and corneal epithelial cell morphology, with associated improvements in tear break-up time and corneal and conjunctival punctate epitheliopathy [74].

The development of topical fenofibrate formulations may further enhance the translational potential of PPARα-targeted therapy in DCN [48]. In diabetic mice, topical fenofibrate improved corneal nerve fiber length and tortuosity coefficient and produced earlier improvements in surface keratopathy compared with oral treatment [48]. Topical administration was also associated with increased neuronal βIII-tubulin mRNA expression in the trigeminal ganglion and elevated tear levels of NGF and SP. Changes in tear biomarkers were not observed with oral treatment [48].

Mechanistically, the neuroprotective effects of fenofibrate appear to involve interconnected neuronal, metabolic, and anti-inflammatory pathways at the ocular surface [27, 48, 74]. Tear proteomic analyses demonstrated upregulation of neurotrophin signaling, neuroactive ligand signaling, mitogen-activated protein kinase pathways, and linoleic acid metabolism following fenofibrate treatment [74]. Fenofibrate also increased levels of glial cell line-derived neurotrophic factor and dimeric brain-derived neurotrophic factor within the corneal epithelium, both of which are implicated in corneal epithelial migration and nerve regeneration respectively [27, 76, 77]. Additional findings suggest that fenofibrate may improve lipid metabolic homeostasis and reduce diabetes-associated triglyceride accumulation linked to corneal nerve degeneration [74]. Gene set enrichment analyses further demonstrated enhancement of sphingolipid metabolism and stem cell pluripotency pathways at the ocular surface, which may contribute to corneal nerve metabolism and epithelial repair [48]. Collectively, these findings suggest that fenofibrate exerts multimodal neuroprotective effects on corneal nerves and epithelium.

Chronic ocular surface inflammation is recognized as an important contributor to DCN progression and impaired epithelial healing [10]. Diabetic corneas exhibit upregulation of inflammatory pathways and mediators at the ocular surface, including adipocytokine signaling, apolipoproteins, serum amyloid A1, TNF-α, NF-κB, interleukin-1 (IL-1), and interleukin-6 [74]. Consistent with the broader anti-inflammatory effects of PPARα activation, both topical and oral fenofibrate reduced ocular surface inflammatory mediators and attenuated corneal immune cell infiltration in diabetic mice and patients with diabetes [35, 48, 74]. These local effects likely involve suppression of corneal NF-κB signaling through both direct PPARα-mediated inhibition and indirect attenuation of TNF-α signaling, a well-established upstream activator of NF-κB [78, 79]. Tear proteomics showed reduced neutrophil-related inflammatory signals increased tear IL-1 receptor antagonist (an endogenous anti-inflammatory mediator) and decreased tear proteins linked to oxidative stress and complement activation, indicating anti-inflammatory and antioxidative effects at the ocular surface [35]. These tear-based molecular changes mapped to modulation of tricarboxylic acid cycle, oxidative phosphorylation, and liver X receptor/retinoid X receptor activation pathways at the ocular surface [35]. *In vivo* confocal microscopy further demonstrated a shift toward a less inflammatory corneal immune cell phenotype following oral fenofibrate treatment [35].

Fenofibrate may additionally modulate ocular surface inflammation through preservation of corneal epithelial mitochondrial integrity *in vitro*, and suppression of ocular cGAS–STING signaling [55]. In experimental models, high glucose and diabetic stress induced mitochondrial dysfunction, increased reactive oxygen species (ROS) production, and promoted mitochondrial DNA release, thereby activating downstream cGAS–STING inflammatory signaling in the corneal epithelium and lacrimal glands [55, 80]. Fenofibrate reversed these changes by upregulating PPARα mRNA expression in corneal epithelial cells, restoring mitochondrial membrane potential, reducing ROS levels, and suppressing corneal expression of cGAS, STING, p-TBK1/TBK1, and p-IRF3/IRF3 [55]. These molecular effects were accompanied by improvements in corneal epithelial healing, tear secretion, tear break-up time, fluorescein staining, and overall ocular surface homeostasis [55]. Together with the findings of Liang et al., [25] these results support mitochondrial dysfunction-driven inflammation and impaired epithelial repair as key therapeutic targets of corneal PPARα agonism.

Table 1 summarizes the current clinical evidence on the use of fenofibrate (PPARα agonism) in DCN and diabetic keratopathy. Collectively, preclinical and preliminary clinical studies suggest that fenofibrate may improve corneal epithelial repair, support corneal nerve regeneration, and reduce ocular surface inflammation in diabetic corneal disease [25, 27, 35, 48, 55, 74]. Topical fenofibrate may be particularly promising because localized ocular delivery produced earlier therapeutic effects than oral administration in preclinical models [48].

**TABLE 1:** Clinical evidence for the use of fenofibrate for treating diabetic corneal neuropathy and diabetic keratopathy.

Authors	Design	Study population	Dose and route (duration)	Main outcomes	Key limitations
Mansoor et al. [35]	Open-label interventional study	Type 2 diabetes (n = 41)	78% 100 mg/day and 22% 300 mg/day, oral (30 days)	• Reduced corneal DC density, area, and elongation/length (suggesting reduced DC activity) • Reduced TNF-alpha, NF-kappaB, complement 4B, and cytochrome B5 • Upregulated anti-inflammatory IL-1 receptor antagonist • Improved corneal epithelial cell density; improved TBUT, corneal staining, and OSDI scores	• Small sample size • Open-label single-arm design • No randomized placebo-treated diabetic control group • Short treatment and follow-up duration (30 days) • No comparator treatment arm • Single-country/specialist-centre recruitment • Dose heterogeneity by renal function (78% on 100 mg, 22% on 300 mg)
Teo et al. [74]	Open-label interventional study	Type 2 diabetes (n = 30)	Same regimen as Mansoor et al.	• Improved CNFD and corneal nerve fiber width (reduced nerve edema) • Enhanced tear SP concentrations (with SP changes associated with CNFD improvement) • Improved corneal epithelial cell circularity • Improved TBUT, corneal and conjunctival staining, and superficial punctate keratopathy • Proteomic upregulation of neurotrophin signaling with suppression of complement cascades and neutrophil reactions	• Small sample size • Open-label single-arm design • No randomized placebo-treated diabetic control group • Short treatment and follow-up duration (30 days) • No comparator treatment arm • Single-country/specialist-centre recruitment • Several corneal nerve parameters showed non-significant trends • Automated nerve analysis may have underestimated nerve parameters

## Dry eye disease

DED is a multifactorial disease of the ocular surface characterized by a loss of homeostasis of the tear film [81]. Tear film instability and hyperosmolarity, ocular surface inflammation and damage, and neurosensory abnormalities also play etiological roles. Clinically, DED is associated with significant ocular discomfort and reduced quality of life. The global burden of DED is also likely to increase with population aging and the growing prevalence of lifestyle-related risk factors, including those affecting young adult and pediatric populations [82].

Corneal epithelial microvilli play an essential role in tear film stability and adherence to the ocular surface, and disruption of microvillar architecture has been implicated in the pathogenesis of DED [83]. In a sleep deprivation-induced DED mouse model, fenofibrate ameliorated microvillar abnormalities and largely restored corneal microvillar density and morphology [83]. Tang et al. proposed that these effects were mediated through corneal PPARα-dependent regulation of lipid metabolism together with increased transient receptor potential cation channel subfamily V member 6 (TRPV6) expression and downstream ezrin phosphorylation in corneal tissues, both of which are important for microvillar structural integrity. Consistent with this mechanism, administration of the PPARα antagonist MK886 in normal mice and cultured corneal epithelial sheets induced dry eye–like changes, including reduced PPARα and TRPV6 expression in corneal epithelial cells and microvilli that were rougher, shorter, and less dense [83]. These findings suggest that corneal PPARα signaling is important for maintaining the corneal epithelial surface and tear film homeostasis [83].

Chronic ocular inflammation is a central contributor to DED pathogenesis [84]. In an obstructive sleep apnea-induced DED mouse model, Wang et al. demonstrated marked inflammatory cell infiltration, NF-κB activation, and abnormal lipid accumulation within lacrimal gland acinar cells [85]. They proposed that chronic intermittent hypoxia upregulated hypoxia-inducible factor alpha (HIFα) in the lacrimal glands, leading to suppression of PPARα signaling and downstream dysregulation of lipid metabolism locally [85]. Reduced PPARα activity in the lacrimal gland was associated with impaired mitochondrial fatty-acid oxidation, increased lipid synthesis and uptake, lipid accumulation in acinar cells, oxidative stress, and and NF-κB-mediated inflammation [85]. Fenofibrate attenuated lipid accumulation, suppressed inflammatory signaling in lacrimal gland tissue, and preserved lacrimal gland function, suggesting that restoration of lacrimal gland PPARα activity may improve both metabolic and inflammatory aspects of DED pathogenesis [85].

Fenofibrate may also exert broader immunomodulatory effects in autoimmune ocular surface disease. In a Sjögren syndrome-like dacryoadenitis mouse model, fenofibrate reduced systemic Th1/Th17 responses and enhanced Treg frequencies in spleen and cervical lymph nodes, while lacrimal glands demonstrated reduced inflammatory infiltration and expression of Th1/Th17-associated cytokines and transcription factors together with increased Foxp3 and TGF-β1 expression, suggesting restoration of local immune homeostasis [49]. The authors proposed that fenofibrate-mediated activation of PPARα upregulated liver X receptor-β signaling, which may contribute to suppression of pathogenic Th1/Th17 polarization while promoting Treg differentiation and function, thereby alleviating autoimmune dacryoadenitis and improving tear secretion and ocular surface integrity [49].

In addition to lacrimal gland effects, fenofibrate appears to directly protect the ocular surface epithelium. He et al. demonstrated that topical fenofibrate attenuated benzalkonium chloride-induced tear film instability, preserved conjunctival goblet cell density, and reduced corneal macrophage infiltration and corneal expression of inflammatory mediators including TNF-α and interleukin-6 [57]. These protective effects were abolished by MK886, supporting a PPARα-dependent mechanism acting locally in the eye. *In vitro* experiments further showed that fenofibrate directly suppressed activated macrophages [57].

In summary, preclinical studies suggest that fenofibrate may attenuate key pathogenic features of DED through corneal anti-inflammatory, metabolic, and immunomodulatory effects, including preservation of corneal epithelial integrity, stabilization of the tear film, and suppression of ocular surface inflammation [49, 57, 83, 85].

## Corneal chemical burns and neovascularization

Chemical burns are among the common causes of corneal NV [86]. Following chemical burns to the cornea, activation of the inflammatory cascade leads to the upregulation of proteolytic enzymes and release of angiogenic factors, which results in the proliferation and migration of vascular endothelial cells into the cornea [86, 87]. These processes ultimately promote corneal NV and loss of corneal transparency [87]. In the United States, ocular surface chemical injuries cost emergency departments roughly $106.7 million over 4 years [88]. Managing corneal NV after chemical burns remains a major challenge in ophthalmology, as current topical treatments do not consistently prevent persistent NV and scarring [17].

Preclinical studies suggest that corneal PPARα plays an important role in regulating early inflammatory and epithelial proliferative processes during corneal wound healing [89]. Following corneal injury, *Ppara* knockout mice demonstrated delayed neutrophil recruitment, impaired inflammatory resolution, and reduced proliferative activity in the cornea compared with wild-type mice, supporting a role for corneal PPARα in coordinating early wound-healing responses after trauma [89].

Following alkali burns, corneal PPARα expression was significantly reduced, particularly within stromal keratocytes, and this reduction was associated with increased corneal NV [90]. Consistent with this, *Ppara* knockout mice developed substantially greater corneal NV after alkali injury together with increased expression of vascular endothelial growth factor receptor 3 (VEGFR3) and matrix metalloprotease 13 (MMP13) in the cornea, two mediators implicated in angiogenic signaling and extracellular matrix remodeling [90]. Additional *in vitro* studies further demonstrated that PPARα deficiency in keratocytes directly increased VEGFR3 and MMP13 expression, supporting a role for stromal PPARα signaling in suppressing keratocyte-driven angiogenic responses after corneal injury [90].

Topical fenofibrate reduced inflammatory cell infiltration in the cornea and suppressed corneal expression of pro-inflammatory mediators including IL-1β, interleukin-6, and monocyte chemoattractant protein-1 in experimental corneal injury models [32, 91]. Mechanistically, these anti-inflammatory effects were associated with modulation of corneal NF-κB signaling, evidenced by reduced nuclear localization of NF-κB and increased NF-κB inhibitor α (IκBα) expression in corneal epithelial cells, consistent with greater cytoplasmic retention and local inhibition of NF-κB activatoin [32, 91, 92]. In parallel, fenofibrate also attenuated corneal NV, as demonstrated by reduced JG-12–positive capillary lumens and lower VEGF-A and angiopoietin-2 expression [50]. In alkali-burned corneas and cultured keratocytes, fenofibrate upregulated PPARα while suppressing corneal VEGFR3 and MMP13 expression, supporting a PPARα-dependent anti-angiogenic mechanism at the stromal/keratocyte level [90]. Additional studies indicate that fenofibrate mitigates corneal oxidative stress and improves corneal fatty acid oxidation, reducing lipid accumulation and, lipid peroxidation-associated injury, and lowering corneal VEGF-A and VEGF-C expression [93]. Collectively, these findings support intertwined local anti-inflammatory, anti-angiogenic, metabolic, and keratocyte-regulatory mechanisms underlying fenofibrate-mediated protection in corneal injury.

Beyond inflammation and angiogenesis, fenofibrate may also modulate stromal fibrosis after chemical injury. Following alkali burns, local inflammatory cytokines promote keratocyte differentiation into α-smooth muscle actin (α-SMA)-positive myofibroblasts, contributing to stromal scarring and opacity through disorganized extracellular matrix deposition [94]. Fenofibrate reduced stromal accumulation of α-SMA-positive myofibroblasts and type III collagen deposition, while treated corneas demonstrated a more organized stromal collagen architecture with less edema and structural disruption than untreated controls [50].

Preclinical studies have also explored strategies to enhance fenofibrate delivery and therapeutic efficacy. In an alkali burn model, co-administration of fenofibrate with the PPARγ agonist pioglitazone produced greater reductions in corneal inflammation and fibrosis than PPARα agonism alone, likely through complementary inhibition corneal NF-κB signaling [91, 92]. In a separate study, incorporation of fenofibrate into a ROS-responsive antioxidant hydrogel enabled sustained drug release and further reduced neovascular vessel density and diameter [95]. More recently, a topical oil-in-water fenofibrate microemulsion eyedrop was developed in a nitrogen mustard–induced corneal injury model [96]. This nanoscale formulation improved corneal drug exposure, was well tolerated *in vitro* and *in vivo*, and, when administered three times daily, significantly reduced vesicant-induced corneal ulceration, NV, and opacity, while preserving epithelial structure and partially restoring epithelial PPARα expression [96]. Collectively, these preclinical findings support the potential of combination approaches and targeted delivery platforms, such as PPARα/PPARγ co-agonism, hydrogels, and microemulsions, to enhance fenofibrate efficacy in corneal NV and chemical injury.

Collectively, these findings support a broader role for PPARα signaling in preserving corneal transparency and regulating wound-healing responses after ocular surface injury.

## Fuchs endothelial corneal dystrophy

FECD is the most common corneal dystrophy and is characterized by progressive, irreversible endothelial cell dysfunction, resulting in corneal edema and the formation of Descemet membrane guttae due to abnormal extracellular deposits [97]. FECD remains a leading indication for corneal transplantation, and there are currently no established disease-modifying treatments [98, 99]. As a result, management of advanced disease often requires surgery, although this approach is limited by donor tissue shortage and the technical complexity of procedures such as Descemet membrane endothelial keratoplasty [98, 99].

Li et al. reported that fenofibrate may mitigate pathological changes relevant to FECD [56]. Fenofibrate appears to exert its effects by modulating metabolic pathways critical for corneal endothelial cell function and fibrosis. Gene set enrichment analysis of the corneal samples from patients with FECD showed downregulation of fatty acid metabolism and mitochondrial fatty acid oxidation, together with impaired PPARα signaling. Consistently, *Ppara* knockout mice exhibited corneal damage and increased fibrosis, supporting an important role for PPARα in maintaining corneal endothelial cell integrity [56]. *In vitro*, fenofibrate attenuated transforming growth factor beta (TGF-β)–induced fibrotic changes and preserved normal corneal endothelial cell morphology [56]. *In vivo*, fenofibrate treatment was associated with preservation of corneal endothelial morphology and reduced corneal edema. Mechanistically, these effects were associated with enhanced corneal fatty acid oxidation, improved mitochondrial homeostasis, and reduced corneal expression of fibrosis markers, including collagen I and α-SMA [56].

## Diabetic retinopathy and diabetic macular edema

DR initially manifests as non-proliferative DR (NPDR), which is characterized by pericyte and endothelial cell injury that leads to microaneurysm formation, disruption of the blood–retinal barrier, and capillary occlusion with consequent retinal ischemia [3, 11]. Retinal ischemia stimulates VEGF expression, driving increased vascular permeability and NV, which mark the transition to proliferative DR (PDR) [11]. DME, a major cause of visual impairment in DR, results from macular thickening due to pathological fluid accumulation [100]. Emerging evidence also suggests that retinal neurodegeneration and glial dysfunction may precede and contribute to microvascular abnormalities traditionally associated with DR [101]. Current treatment options, such as intravitreal anti-VEGF injections and laser photocoagulation, are effective in selected setting but remain invasive, costly, and treatment-intensive, which may limit real-world feasibility [16]. In addition, persistent edema remains common in a proportion of eyes with DME despite anti-VEGF therapy [15], and broader inflammatory and neurodegenerative mechanisms of the disease remain insufficiently addressed.

DR and DME are characterized by chronic inflammation, oxidative stress, leukostasis, neurodegeneration, and breakdown of the blood-retinal barrier [3, 11, 100]. Preclinical studies suggest that PPARα activation exerts broad retinal protective effects by stabilizing the blood-retinal barrier, preserving pericyte and endothelial cell function, and suppressing inflammatory signaling pathways implicated in retinal vascular leakage and ischemia [30, 102]. A central mechanism appears to involve inhibition of NF-κB signaling through restoration of sirtuin-1 (SIRT1), which deacetylates the p65 NF-κB subunit, as as evidenced by increased SIRT1 and reduced NF-κB in the rat retina *in vivo*, and by PPARα-dependent, SIRT1-mediated NF-κB suppression in retinal endothelial cells *in vitro* [103, 104]. *In vitro*, fenofibrate mediated suppression of NF-κB is abolished following PPARα inhibition or SIRT1 silencing in human retinal endothelial cells, supporting a PPARα–SIRT1–NF-κB regulatory axis [104]. In type 1 diabetic models, suppression of NF-κB activation in the retina is associated with reduced retinal expression of downstream inflammatory mediators and adhesion molecules, including monocyte chemoattractant protein-1 and intercellular adhesion molecule-1 (ICAM-1) [29]. Consistent with these preclinical findings, clinical studies have reported lower circulating and intraocular levels of pro-inflammatory cytokines following fenofibrate treatment [105].

Retinal leukostasis is another important contributor to capillary non-perfusion and ischemia in DR [3]. Diabetes-induced downregulation of PPARα in monocytes promotes mitochondrial dysfunction and activation of the cGAS–STING pathway, leading to enhanced endothelial adhesion, migration, phagocytosis, and inflammatory activation of monocytes *in vitro* and *ex vivo* [28]. Fenofibrate via PPARα activation, preserves monocyte mitochondrial integrity, reduces cytosolic mitochondrial DNA release, suppresses monocyte cGAS–STING signaling, and, *in vivo*, thereby attenuates retinal leukostasis in diabetic models [28]. Consistent with these anti-inflammatory effects, fenofibrate also reduces retinal oxidative stress, a key driver of VEGF upregulation, NF-κB activation, and retinal vascular dysfunction in DR [72], in part through activation of Nrf2-dependent antioxidant pathways and suppression of NLRP3 inflammasome signaling in diabetic mouse retinas. It further attenuates Nox-mediated oxidative stress and Wnt/β-catenin signaling in the retina and retinal pigment epithelium, thereby reducing retinal inflammation and vascular leakage [106, 107].

Fenofibrate also exerts anti-angiogenic effects relevant to advanced DR and DME. Through retinal PPARα-dependent mechanisms, fenofibrate suppresses retinal VEGF expression, likely in part through inhibition of pathological HIF-1α signaling in the retina of ischemic and diabetic models [29, 108]. Suppression of retinal NF-κB signaling may represent an additional pathway contributing to reduced VEGF expression and downstream angiogenic activity in the diabetic retina [29, 109]. In parallel, both fenofibrate and FA also demonstrate PPARα-independent anti-angiogenic effects through inhibition of CYP2C-mediated production of the pro-angiogenic metabolite 19,20-epoxydocosapentaenoic acid (19,20-EDP), evidenced by reduced plasma 19,20-EDP *in vivo* and reversal of FA mediated inhibtion of angiogenesis *ex vivo* and in retinal enodethial cells by 19,20-EDP [38].Collectively, these findings suggest that fenofibrate and FA may inhibit retinal angiogenesis through multiple complementary local inflammatory, hypoxia-responsive, and lipid-metabolic pathways.

Preservation of blood-retinal barrier integrity by FA in experimental models may constitute another major component of the retinal protective effects of fenofibrate. In RPE cells, IL-1β induces NF-κB activation, inflammatory mediator release, tight junction disorganization, and hyperpermeability through AMPK-dependent pathways [39, 110]. FA suppresses these changes in a PPARα-dependent manner while also reducing IL-1β-induced AMPK phosphorylation [39, 110]. FA also protects the outer blood-retinal barrier dysfunction through AMPK-dependent, PPARα-independent mechanisms, reducing oxidized LDL-induced adhesion molecule and VEGF expression [111]. It limits high-glucose-induced extracellular matrix accumulation and COX-2 upregulation in retinal cells [112, 113]. Given that COX-2 promotes inflammatory signaling and regulates VEGF expression through the COX-2/prostaglandin E2 pathway, its suppression by FA likely contributes to barrier protection through both direct anti-inflammatory effects and reduced VEGF-driven vascular permeability [112, 114].

Beyond vascular protection, FA exerts direct cytoprotective effects, while fenofibrate provides neuroprotection effects within the diabetic retina. FA attenuates ROS production and stress-activated kinase signaling in RPE cells under hypoxic and hyperglycemic conditions while enhancing insulin-like growth factor-1 receptor-mediated survival signaling and autophagy-related pathways [115]. Fenofibrate suppresses Müller cell gliosis, reduces nerve fiber layer edema, preserves retinal function, and mitigates diabetes-induced microglial activation and neuroinflammation in the retina [24, 103, 106]. These effects appear closely linked to microglial PPARα, which improves mitochondrial function and limits a glycolytic shift under diabetic stress, thereby reducing oxidative stress and suppressing STING-associated inflammatory signaling in retinal microglial [24, 116].

Recent advances in ocular drug delivery further support continued investigation of fenofibrate in retinal disease. Fenofibrate-loaded biodegradable poly(lactic-co-glycolic acid) (PGLA) nanoparticles achieved sustained intraocular drug delivery and reduced vascular leakage, leukostasis, VEGF expression, and NV in preclinical diabetic and choroidal NV models without obvious retinal toxicity [117]. More recently, long-acting PLGA fenofibrate microparticles with high drug loading and ≥6-month intravitreal release after a single injection preserved ERG function, reduced leukostasis and albumin extravasation, and increased retinal PPARα expression in streptozotocin-induced diabetic rats, with sustained retinal drug levels and acceptable safety in rabbits [118]. Collectively, these preclinical data suggest that retinal-targeted sustained-release fenofibrate formulations may improve ocular bioavailability, extend dosing intervals, and broaden fenofibrate’s application in DR, although clinical data are not yet available.

Across randomized and observational studies, fenofibrate appears to act as an adjunctive disease-modifying therapy primarily in eyes with pre-existing mild-to-moderate DR, with limited evidence of benefit in eyes with no DR or advanced disease at baseline [42, 44, 46].

In FIELD, fenofibrate significantly reduced a composite imaging outcome (2-step ETDRS progression or new macular edema or retinal laser treatment) and the need for retinal laser as an independent outcome; imaging data also support a reduction in ETDRS step progression [44]. ACCORD Eye showed that fenofibrate significantly reduced ≥3-step ETDRS progression, both when tested independently and when combined in a composite with retinal laser or vitrectomy [42]. In both FIELD and ACCORD Eye, these benefits were largely confined to participants with mild-to-moderate NPDR and were not clearly seen in those with severe or no retinopathy at baseline [42, 44]. Follow-up data from ACCORDION indicated that these retinal protective benefits were not sustained 3–5 years after discontinuation of fenofibrate, implying that ongoing therapy may be required to maintain disease-modifying effects [119].

The LENS trial, which enrolled individuals with non-referable, early DR or maculopathy (approximately corresponding to early ETDRS levels), demonstrated a significant reduction in “any” retinopathy or maculopathy progression and in progression to referable DR or maculopathy (the latter as a composite with retinopathy/maculopathy treatment) [46]. Notably, LENS did not find a statistically significant reduction in treatment alone (intravitreal injection, retinal laser, or vitrectomy) when analysed as an independent outcome [46].

Overall, there is little evidence that fenofibrate improves visual acuity, visual function, or quality of life over the follow-up periods studied [42, 44, 46]. Formal assessment in LENS found no significant or clinically meaningful benefit in any of these parameters [46].

Two large cohort studies echoed these trial findings. A Korean study showed a significant reduction in a composite DR-progression outcome (vitreous hemorrhage, vitrectomy, retinal laser, intravitreal injections, retinal detachment) only in patients with baseline DR, with no significant benefit in those without DR [120]. When components were analysed individually, vitreous hemorrhage, retinal laser, and intravitreal injections remained significantly reduced, whereas vitrectomy and retinal detachment did not [120]. A US claims-based study in patients with NPDR at baseline reported significantly lower progression to vision-threatening DR (PDR or DME) and to PDR independently, but no significant effect on incident DME considered alone, suggesting that the reduction in vision-threatening DR was driven mainly by decreased progression to PDR [121].

Across these five trials, most fenofibrate exposure occurred in the context of concomitant statin therapy, and the strongest retinal benefits were demonstrated on a fenofibrate-plus-statin background [42, 44, 46, 120, 121]. Consequently, evidence for fenofibrate as monotherapy in DR is limited, and current data primarily support its role as add-on treatment to statin therapy.

A secondary analysis of FIELD suggested that the magnitude of fenofibrate’s protective effect may vary according to baseline haptoglobin concentration, with the greatest benefit observed in individuals with the lowest baseline haptoglobin levels [122]. Additional clinical studies suggest that fenofibrate may improve retinal microvascular health and enhance endogenous vascular repair mechanisms [123, 124]. A retrospective cohort study from Taiwan further reported that regular fenofibrate use was associated with a reduced risk of new-onset retinopathy in patients without baseline DR, although potential underdiagnosis within administrative datasets limits interpretation [125].

By contrast, the clinical role of fenofibrate in DME remains less consistent [42, 44, 46, 120, 121, 126, 127]. LENS demonstrated a clear benefit of fenofibrate on DME-related outcomes in people with early, non-referable disease: incident macular edema (any centre- or non-centre-involving macular edema) was significantly reduced, and referable maculopathy (exudates or blot haemorrhages within 1 disc diameter of the fovea) was also significantly reduced when measured independently [46]. By contrast, MacuFEN, which treated eyes with established, predominantly centre-involving DME that did not require immediate treatment and moderate-to-severe DR, found no significant advantage of FA over placebo on OCT-measured macular thickness/volume, visual acuity, or need for ocular treatment over 12 months [127].

Notably, in other trials where fenofibrate significantly reduced the need for treatment, “treatment” encompassed procedures for both DR and maculopathy; these findings indirectly suggest a reduction in treatment for DME, but interpretation is limited because treatment was not reported separately for DR vs. DME and no explicit proportional breakdown was provided [42, 44, 120]. Similarly, although FIELD reported a significant reduction in a composite imaging outcome that included new macular edema, interpretation is limited because new macular edema was not analysed as an isolated endpoint and the relative contribution of each component within the composite is unclear [44]. When analysed as an individual outcome, no significant effect on incident DME was reported by the US cohort trial mentioned above [121].

A single-centre randomized controlled trial provided evidence that adjunctive fenofibrate may enhance mean reduction in central macular thickness after DME treatment (anti-VEGF, intravitreal triamcinolone, grid/focal macular laser, and/or pan-retinal photocoagulation) [126]. However, this is limited by potentially unequal and unreported distributions of these treatments between groups, making it difficult to attribute between-group differences solely to fenofibrate, and by small sample size and short treatment duration and follow-up, which reduce statistical power [127]. Consistent with LENS and MacuFEN, no significant visual acuity benefit was detected over placebo or control groups [46, 126, 127].

In summary, preclinical evidence supports fenofibrate/FA as retinal protective agents that modulate inflammation, leukostasis, neurodegeneration, and blood-retinal barrier integrity [28, 29, 38, 39, 102–104, 106, 107, 110–113, 115, 116]. Randomized and observational data support fenofibrate (mainly as adjunct to statins) as a disease-modifying therapy that slows progression of mild–moderate pre-existing DR, reduces the development of PDR and, in some studies (notably LENS), incident DME, and may lower the need for retinal interventions, with little evidence of benefit in eyes with no DR, advanced DR, or for improving visual acuity, function, or quality of life [42, 44, 46, 120, 121]. Evidence for a therapeutic effect in established, centre-involving DME is generally not supportive; indirect signals that fenofibrate may reduce maculopathy-related treatment or modestly improve central macular thickness are weak and constrained by heterogeneous co-treatments and other trial-specific limitations [42, 44, 120, 126, 127]. Overall, current clinical conclusions for DME are strongest for fenofibrate as an add-on preventive therapy in early DR rather than as treatment for chronic or advanced DME. Table 2 summarizes the clinical evidence for the treatment of DR and DME with fenofibrate. Novel drug delivery systems, such as fenofibrate-loaded biodegradable nanoparticles, offer better ocular bioavailability and sustained release, but remain experimental at this stage [117, 118].

**TABLE 2:** Summary of clinical evidence for the treatment of DR and DME with fenofibrate.

Authors	Design	Study population	Dose and route (duration)	Main outcomes	Key limitations
ACCORDION eye study [119]	Post-RCT observational follow-up of ACCORD	Type 2 diabetes, established CVD, prior ACCORD participants (n = 1,310)	As in parent ACCORD trial	• Fenofibrate benefit on DR progression did not persist after trial end (OR 1.13, p = 0.60) • Intensive glycemic control showed persistent benefit on DR progression (5.8% vs. 12.7%; OR 0.42, p < 0.0001) • Strict blood pressure control had no effect	• Low recruitment/retention • Unequal demographic spread in returning participants • Retinopathy data collected only once (at year 4) • HbA1c and lipid differences between treatment groups diminished after trial ended
Keech et al. (FIELD) [44]	Multicentre, multinational, double-blind, placebo-controlled RCT	Type 2 diabetes, age 50–75 (n = 9,795; fenofibrate 4,895, placebo 4,900). Ophthalmology substudy: n = 1,012	Fenofibrate 200 mg/day vs. placebo (∼5 years)	• Laser treatment reduced by 31% overall (HR 0.69, 95% CI 0.56–0.84, p = 0.0002; ARR 1.5%): 31% reduction for macular edema (p = 0.002) and 30% for proliferative DR (p = 0.015) • In the substudy, 2-step progression non-significant overall but significantly reduced in patients with pre-existing DR (3.1% vs. 14.6%; p = 0.004) • No effect on visual acuity. Benefit independent of plasma lipid levels	• Laser treatment was a tertiary endpoint (not primary) • Retinal photography not done in all patients at baseline • Substudy underpowered for 2-step progression (observed placebo rate ∼12% vs. expected ∼25%, yielding ∼36% power) • More placebo patients commenced statins (17% vs. 8%), potentially diluting the observed effect • No statin co-administration at baseline limits relevance to current standard-of-care practice
Bonora et al. [124]	Phase IV RCT, single-blind, placebo-controlled	Adults with DR (n = 42), diabetes duration ∼18 years	Fenofibrate 145 mg/day vs. placebo (12 weeks)	• Fenofibrate significantly increased circulating HSPCs (CD34+/CD133+) vs. placebo, projecting reduced DR progression risk • No effect on endothelial progenitor cells or inflammatory markers	• Small sample size, short treatment duration, and single-centre design • Retinopathy benefit estimated indirectly (HSPC surrogate) rather than observed clinical outcomes; vascular and hematopoietic function of HSPCs not evaluated; exclusion of CKD, advanced liver disease, and other comorbidities
ACCORD eye study [43]	Multicentre RCT substudy	Type 2 diabetes at high CVD risk (n = 2,856)	As in parent ACCORD trial	• Fenofibrate significantly reduced DR progression (three-step, photocoagulation, or vitrectomy) at 4 years (OR 0.60, p = 0.0056) • Greatest benefit in patients with mild baseline retinopathy (OR 0.27, p = 0.0009) • Additive effect with intensive glycemic control	• Retinopathy assessed from fundus photos at only two time points (baseline and year 4) • 17.7% lost to follow-up • Participants with missing data had less favourable baseline risk profiles
ACCORD eye study – additional ocular outcomes [42]	Multicentre RCT substudy (companion paper to Ref. 43)	Same trial population as above; same n = 2,856	As in parent ACCORD trial	• Fenofibrate significantly slowed DR progression, especially in mild baseline retinopathy • Additive effect observed with strict glycemic control and fenofibrate combined	• Retinopathy assessed from fundus photos at only two time points • Did not include participants with previously treated proliferative DR, limiting applicability to advanced disease • Some subgroup analyses were exploratory/*post hoc* • Composite endpoint combined photographic progression with treatment-based events
Ju et al. [105]	Non-randomized controlled interventional study	Type 2 diabetes with NPDR or PDR (n = 160 intervention + control), plus 30 with no DR	Fenofibrate 160 mg/day + conventional therapy (12 weeks)	• DR patients had higher serum IL-1-beta, TNF-alpha, VEGF, Lp-PLA2 than non-DR controls • Fenofibrate significantly decreased these cytokines in both NPDR and PDR	• Small sample size, short treatment duration, and single-centre design • Treatment allocation based on patient acceptance (non-randomized) • No clear blinding • No DR progression assessment • Limited details of background therapies • Baseline disease duration differed across groups • Serum biomarkers may not reflect intraocular retinal inflammation
Kim et al. [120]	Retrospective observational cohort (propensity-matched)	Type 2 diabetes + metabolic syndrome, statin users (n = 22,395 fenofibrate + statin vs. 43,191 statin-only)	Median on-treatment follow-up 37.2 months	• Fenofibrate associated with reduced DR progression (mainly in patients with pre-existing DR), reduced risk of vitreous hemorrhage, laser photocoagulation, and intravitreal injection	• Lack data on medical adherence • No retinal imaging or grading scales • Progression inferred from diagnostic/procedure codes • Unmeasured confounders may remain • Specific intravitreal drugs not identified
Lin et al. [125]	Retrospective population-based cohort study	Type 2 diabetes without baseline DR (n = 2,500 fenofibrate users vs. 29,753 non-users)	Fenofibrate 200 mg/day (3 months to >2 years)	• Fenofibrate associated with reduced incident DR and reduced need for laser treatment • Suggests long-term fenofibrate may prevent new-onset DR	• DR diagnosis based on ICD-9 codes rather than retinal photos • No routine retinal screening (possible under-diagnosis) • Fenofibrate users differed from non-users at baseline • Medication exposure inferred from claims, not adherence-confirmed
Meer et al. [121]	Retrospective observational cohort	Adults >=18 years with NPDR (n = 5,835 fenofibrate users vs. 144,417 non-users)	Not reported	• Fenofibrate decreased risk of vision-threatening DR (HR 0.92, p = 0.01) and PDR (HR 0.76, p = 0.001) • No significant effect on DME alone (HR 0.96, p = 0.27)	• No direct access to clinical examination or retinal imaging • DR/DME outcomes defined by diagnosis/procedure codes with potential outcome misclassification • No detailed dose, adherence, or duration data
Ong et al. [122]	Secondary analysis of RCT data (FIELD)	Adults with type 2 diabetes, Australasian FIELD participants (n = 8,047)	As in parent FIELD trial	• Fenofibrate reduced risk of sight-threatening DR by 32% overall • Greatest benefit in patients with lowest baseline haptoglobin levels • All HP phenotypes benefited	• Only Australian and New Zealand FIELD participants included • Baseline PDR and DME data not available for the full cohort • HP level may reflect broader inflammation, oxidative stress, or liver function rather than a specific retinopathy mechanism
Preiss et al. [46]	National multicentre RCT, double-blind, placebo-controlled	Adults with non-referable DR or maculopathy (n = 1,151; 576 fenofibrate, 575 placebo)	Fenofibrate 145 mg/day (or every other day for reduced renal function); median 4 years	• Fenofibrate significantly reduced progression to referable DR or maculopathy (HR 0.74; 95% CI 0.61–0.90; 32.1% vs. 40.2%) • Reduced macular edema development (HR 0.50; 95% CI 0.30–0.84; 3.8% vs. 7.5%) • No effect on visual function, quality of life, or visual acuity	• Fenofibrate reduced eGFR by ∼8 mL/min/1.73m2 on average • NHS scotland grading is less granular than ETDRS, with no direct mapping between grading systems • Single-field 45-degree macula-centred photos (less retinal coverage than ETDRS 7-field) • OCT not routinely performed (some macular edema may have been missed)
Quinn et al. [123]	Secondary substudy analysis of RCT (FIELD)	FIELD trial participants with gradable retinal images (n = 208)	As in parent FIELD trial	• Fenofibrate significantly reduced central retinal venule caliber at 2 years • Retinal arteriolar metrics unchanged • Suggests fenofibrate may improve retinal microvascular health	• Only 208 of 1,012 FIELD ophthalmology substudy participants had gradable images at both time points (substantial attrition) • Follow-up limited to 2 years while FIELD retinal benefits emerged over longer follow-up • Vessel caliber changes were not associated with ETDRS scores or retinopathy severity change
Massin et al. (MacuFen) [127]	Multicentre RCT, double-blind, placebo-controlled	Adults with DME not requiring immediate treatment (n = 110)	Fenofibrate 135 mg/day (12 months)	• Modest decrease in total macular volume after fluorescein angiography favouring fenofibrate but not statistically significant between groups	• Small sample size, short treatment duration, and single-centre design • No significant between-group differences for visual acuity, ETDRS grading, macular edema grading, hard exudates, or eye procedures • 12-month follow-up may be too short to detect visual/structural effects
Srinivasan et al. [126]	Prospective RCT (coin-toss randomization, unmasked)	Adults with DME (n = 50 patients, 53 eyes)	Fenofibrate 160 mg/day + standard DME protocol (6 months)	• Fenofibrate enhanced reduction in central macular thickness • Non-significant trend toward greater visual acuity improvement • Benefit independent of triglyceride levels or hypertension	• Small sample size, short treatment duration, and single-centre design • Coin-toss randomization with no masking/blinding • Both eyes included from some patients (correlated data) • Baseline characteristics unbalanced • Treatment heterogeneity • Time-domain OCT used (instead of spectral-domain)

## Neovascular age-related macular degeneration

AMD is the leading cause of irreversible vision loss among older adults and is typically classified as dry and wet forms [4]. Neovascular age-related macular degeneration (nAMD), also referred to as wet AMD, is a late and aggressive form of AMD that affects 10%–15% of patients with AMD [12]. It is characterized by the development of pathological choroidal NV, in which abnormal blood vessels grow from the choroid through Bruch’s membrane into the subretinal space [12]. These vessels leak blood and fluid into the subretinal space or into the retina [12]. Current management for nAMD relies predominantly on intravitreal anti-VEGF therapy, which is costly and requires frequent injections, carrying cumulative risks related to repeated intravitreal procedures and potential systemic adverse effects [128].

Preclinical studies suggest that FA suppresses choroidal NV through both PPARα-dependent and PPARα-independent mechanisms [26, 38]. In *Vldlr* knockout mice and laser-induced choroidal NV models, FA reduced choroidal and retinal NV together with vascular leakage, retinal leukostasis, and expression of VEGF, TNF-α, and ICAM-1 in posterior ocular tissues [26]. These PPARα-dependent effects are consistent with the broader anti-inflammatory actions of fenofibrate, particularly NF-κB inhibition, which likely contributes to its local anti-angiogenic and vascular stabilizing activity [26, 129, 130].

In parallel, fenofibrate has also been shown to inhibit choroidal NV in CYP2C8-overexpressing mice, and FA suppresses angiogenesis in a choroidal sprouting assay. Both effects appear to be mediated, at least in part, by a PPARα-independent mechanism involving CYP2C-mediated lipid metabolism [38]. Low-dose fenofibrate reduces plasma 19,20-EDP levels in CYP2C8-overexpressing mice, consistent with CYP2C inhibition, and exogenous 19,20-EDP reverses these anti-angiogenic effects. Together, these findings support a model in which low dose fenofibrate suppresses choroidal NV by inhibiting CYP2C and lowering 19,20-EDP levels [38].

Beyond angiogenesis, both fenofibrate and FA have demonstrated anti-fibrotic effects relevant to late-stage nAMD [36]. In immortalized rat retinal Müller cells*,* FA reversed TGF-β2–induced upregulation of collagen I and connective tissue growth factor and attenuated TGF-β2–induced increases in Wnt signaling components [36]. In *Vldlr* knockout mice, systemic fenofibrate attenuated subretinal fibrosis through inhibition of TGF-β–Smad2/3 and Wnt signaling pathways, while reducing Müller cell activation and retinal expression of collagen I, fibronectin, vimentin, and connective tissue growth factor [36]. Consistent with these molecular changes, immunofluorescene showed reduced staining of fibrotic markers (α-SMA, vimentin, and collagen-1), and protein levels of fibronectin, vimentin, and collagen-1 in 7-month-old *Vldlr* knockout mice retinas [36]. Together, these findings suggest that fenofibrate and FA therapy may not only suppress neovascular activity but also limit fibrotic stages associated with advanced disease.

Recent studies have also explored ocular delivery strategies to improve fenofibrate bioavailability and therapeutic durability in nAMD [51, 117, 118, 128]. Fenofibrate-loaded biodegradable PGLA nanoparticles effectively suppressed choroidal NV and vascular leakage in both laser-induced and *Vldlr* knockout models, with efficacy comparable to daily systemic administration [117]. Sustained-release biomaterial scaffolds targeting the RPE have similarly demonstrated favorable biocompatibility and release characteristics [51]. Long-acting fenofibrate PLGA microparticles provide ≥6-month intravitreal release after a single injection and, in Vldlr−/− and Abca4^−/−^/Rdh8^−/−^ models, reduced retinal and choroidal NV, vascular leakage, and retinal degeneration while improving mitochondrial markers, with sustained posterior segment exposure and no overt ocular or systemic toxicity in rabbits [118]. To overcome the limitations of invasive intraocular delivery, Huang et al. developed a fenofibrate nano-emulsion eye drop that achieved significantly higher retinal, choroidal, and corneal fenofibrate and FA levels than systemic administration while suppressing retinal inflammation, vascular leakage, and laser-induced choroidal NV [128]. Together, these preclinical findings highlight sustained-release intravitreal systems, RPE-targeted scaffolds, and topical nano-formulations as promising strategies to enhance the durability and safety of fenofibrate therapy in AMD.

In summary, preclinical evidence suggests that fenofibrate may suppress key pathogenic processes in nAMD, including choroidal NV, vascular leakage, and subretinal fibrosis [26, 36, 38]. These effects appear to involve both PPARα-dependent anti-inflammatory and anti-fibrotic mechanisms as well as PPARα-independent inhibition of CYP2C-mediated pro-angiogenic signaling [26, 36, 38]. Emerging ocular delivery strategies, including nanoparticles and topical nano-emulsion formulations, further support the translational potential of fenofibrate by improving retinal drug delivery and reducing treatment burden [51, 117, 118, 128].

## Central retinal artery occlusion

Central retinal artery occlusion (CRAO) is an ophthalmic emergency caused by the acute obstruction of retinal blood flow. Patients typically present with sudden, painless vision loss [131]. Its risk factors and demographic profile closely mirror those of ischemic stroke and cardiovascular disease [131]. Given the retina’s high vascularity and extreme sensitivity to ischemia, retinal ganglion cells sustain irreversible damage rapidly if prompt intervention is not provided [132].

Preclinical studies suggest that fenofibrate and FA may exert neuroprotective effects in retinal ischemic injury, including experimental models of CRAO and ischemia–reperfusion injury [31, 37]. In adult mouse models of CRAO, systemic fenofibrate attenuated ischemia-induced retinal dysfunction, as demonstrated by preservation of electroretinography a- and b-wave amplitudes, together with partial preservation of synaptophysin expression, supporting protection of retinal synaptic integrity [37]. Mechanistically, these effects were associated with activation of downstream PPARα target genes in the liver, increased circulating fibroblast growth factor 21 (FGF21), and modulation of hypoxia-responsive retinal pathways, including reduced expression of HIF-1α and Bcl-2/adenovirus E1B 19-kDa interacting protein 3 in the retina, alongside increased retinal glucose transporter 1 expression [37]. These findings are consistent with improved metabolic adaptation to ischemic stress: circulating FGF21 has been linked to neuroprotection through attenuation of neuroinflammation and preservation of retinal photoreceptors, whereas upregulation of glucose transporter 1 in the retina may help sustain glucose uptake and ATP generation during hypoxia [133–135].

Further mechanistic studies demonstrated that PPARα expression is significantly reduced in oxygen–glucose deprivation-treated retinal cells and in ischemia–reperfusion model retinas, particularly within the retinal ganglion cell layer, whereas FA restored PPARα expression both *in vitro* and *in vivo* [31]. FA attenuated ischemia–reperfusion-induced retinal ganglion cell loss, reduced ganglion cell complex thinning, and improved retinal electrophysiological responses [31]. These neuroprotective effects were accompanied by reduced glial fibrillary acidic protein and COX-2 expression, suggesting attenuation of glial activation and retinal inflammation [31].

In summary, preclinical evidence indicates that fenofibrate confer protective effects in retinal ischemic injury relevant to CRAO through PPARα-dependent mechanisms and downstream retinal actions. In the first model, fenofibrate primarily activates PPARα in the liver, increasing circulating FGF21, which in turn modulates retinal hypoxia-responsive pathways and metabolic adaptation [37], whereas in ischemia-reperfusion models FA restores PPARα expression within the retina itself, leading to suppression of glial activation and neuroinflammation and direct neuroprotection of retinal ganglion cells [31].

## Discussion

This review highlights the evolving ophthalmic relevance of fenofibrate beyond its traditional role as a lipid-lowering agent. Interest was initially driven by the FIELD and ACCORD Eye studies, which demonstrated that fenofibrate reduced the progression of DR and the need for retinopathy treatment in patients with mild-to-moderate NPDR [42–44], but accumulating evidence now suggests broader therapeutic potential across both corneal and retinal diseases [27, 35–37, 48–51, 56, 75]. The strength of evidence varies substantially by indication. DR and DME currently have the strongest evidence base, supported by both randomized and non-randomized clinical trials and translational studies [42–44, 46, 105, 119–122, 124–127]. Clinical evidence for DCN remains preliminary but promising [35, 74], whereas data for DED, corneal burns and NV, FECD, nAMD, and retinal ischemic injury remain preclinical [26, 31, 55, 56, 93]. Fenofibrate therefore occupies different stages of translational maturity across ophthalmology, ranging from clinically supported slowing of DR progression and reduction in treatment-requiring disease to exploratory mechanistic investigation in other ocular diseases.

One of the most consistent effects of fenofibrate across disease models was preservation of mitochondrial integrity and metabolic function in ocular tissues and relevant systemic cell types [24, 25, 28, 35, 55, 56, 85, 102, 116]. *PPARα* knockout models conversely show mitochondrial dysfunction and impaired cellular homeostasis across both corneal and retinal tissues [24, 25, 28, 89, 116]. Fenofibrate likely enhances mitochondrial biogenesis and function via canonical PPARα–PGC-1α signaling, upregulating NRF-1 and mitochondrial transcription factor A (TFAM) to support mitochondrial DNA maintenance and respiration, as demonstrated in non-ocular systems and plausibly operating in ocular tissues as well [136]. Improved mitochondrial function may increase fatty-acid oxidation, reduce intracellular lipid accumulation and lipotoxicity, and downstream local inflammatory signaling, which may help explain impaired epithelial healing in diabetic and alkali-burned corneas given the high ATP demand of repair and tissue regeneration [25, 55, 137].

Preservation of mitochondrial integrity may also underlie many of fenofibrate’s anti-inflammatory effects. Dysfunctional mitochondria promote ROS accumulation and reduced fatty-acid oxidation, amplifying oxidative stress, NF-κB activation, inflammatory cytokine production, and VEGF-associated angiogenic signaling [138–141]. By restoring mitochondrial health in both ocular cells, such as corneal epithelium and retinal microglia, and systemic immune cells, including monocytes, fenofibrate may reduce ROS generation, limit mitochondrial DNA release and cGAS–STING activation, and thereby decrease leukostasis, vascular leakage, and inflammation, contributing to reduced immune-cell infiltration, inflammatory mediator expression, and NV across multiple models. In diabetic mouse retinas, fenofibrate activates Nrf2 and downstream antioxidant pathways such as NQO-1 and HO-1 [105], and because Nrf2 regulates mitochondrial biogenesis, respiration, and mitophagy [142], crosstalk between PPARα and Nrf2 may further couple mitochondrial protection with suppression of oxidative stress and NF-κB-mediated inflammation [143].

Anti-fibrotic effects emerged across corneal burn, nAMD, and FECD disease models, with reduced α-SMA–positive myofibroblasts, collagen deposition, stromal fibrosis, and subretinal fibrosis, associated with inhibition of TGF-β–Smad2/3 and Wnt signaling [36, 50, 56]. This is particularly relevant in corneal injury, where TGF-β drives keratocyte-to-myofibroblast differentiation and stromal scarring [144]. In contrast, increased TGF-β activity in a Sjögren syndrome–like dacryoadenitis model (affecting lacrimal glands and systemic lymphoid tissues) may have supported Treg-mediated immune regulation [49, 145], suggesting context-dependent modulation of TGF-β signaling. Collectively, these findings indicate that fenofibrate functions less as a single-target drug and more as a pathway-modifying agent that can interrupt interconnected cycles of mitochondrial dysfunction, oxidative stress, inflammatory amplification, angiogenesis, and fibrosis across multiple ophthalmic diseases.

A major unresolved question is whether fenofibrate should be positioned primarily as an early disease-modifying therapy or as a treatment for advanced ophthalmic disease. Across DR studies, the most consistent evidence supports a role in slowing early progression rather than reversing established pathology, with FIELD, ACCORD Eye, and LENS indicating that benefit is greatest in patients with mild-to-moderate pre-existing DR and limited in those without baseline retinopathy or with advanced disease [42, 44, 46]. This is reinforced by retrospective studies showing benefit in patients with NPDR [121], but no significant benefit in those without baseline retinopathy [120]. Preliminary clinical data in DCN similarly show improvements in corneal nerve and ocular surface parameters in patients with abnormal baseline findings, but the efficacy of fenofibrate in established DCN and diabetic keratopathy remains uncertain [35, 74].

This stage-dependent pattern is particularly evident in DME. The apparent discrepancy in findings between LENS and MacuFEN likely reflect differences in follow-up duration, sample size, outcome measures, and, importantly, baseline disease severity [46, 127]. In LENS, beneficial outcomes were seen in a population in which the majority had no baseline maculopathy (90%), whereas MacuFEN showed no significant treatment benefit in participants with established, treatment-eligible DME and more advanced DR [46, 127]. Its anti-inflammatory and vascular-stabilizing effects may limit microvascular deterioration and reduce DME development, but may be insufficient to consistently resolve established center-involving edema or improve visual acuity [126, 127].

Together, these findings support fenofibrate primarily as an early progression-modifying adjunct to existing therapies rather than as a treatment for advanced ocular disease. Outside DR, the optimal treatment window remains uncertain, and late-stage efficacy rests mainly on limited preclinical evidence. Preclinical data in nAMD, in which fenofibrate and FA reduced subretinal fibrosis and profibrotic signaling in late-stage models [36], suggest potential activity in fibrotic disease but remain hypothesis-generating and do not outweigh the stronger clinical signal favoring earlier intervention.

Another key translational issue is the durability of fenofibrate’s benefit after treatment discontinuation. In ACCORDION, the protective effect on DR progression observed during ACCORD was not sustained once fenofibrate was stopped [119], and outside DR, studies have not evaluated outcomes after cessation [35–37, 50, 56, 85, 128], leaving it unclear whether benefits persist off therapy. If continuous long-term administration is required to maintain efficacy, fenofibrate may be less attractive as a “low-intensity” alternative.

Nevertheless, long-term adjunctive oral fenofibrate could still lessen overall patient burden if it slows progression to sight-threatening disease, reduces complications, or decreases the need for invasive treatment. In DR, the strongest evidence supports a significant reduction in retinal laser treatment with fenofibrate [42, 44, 120, 125]. Effects on vitrectomy and intravitreal injections are less consistent, with both significant and non-significant benefits reported [42, 46, 120]. Most notably, LENS found no significant effect on a composite treatment outcome (intravitreal injection, retinal laser, vitrectomy), likely because it enrolled a much earlier, lower-event-rate population and was primarily powered to detect differences in referable disease and incident macular edema [46]. Further long-term studies are needed to clarify whether fenofibrate should be used indefinitely, intermittently, or at specific disease stages, and whether adjunctive therapy meaningfully reduces DR complications and invasive interventions beyond standard care alone.

Ocular drug delivery is another key translational consideration. Oral fenofibrate has the strongest clinical evidence, particularly in DR, but systemic therapy carries renal, hepatic, and muscle-related safety considerations, especially in patients with diabetes, renal impairment, or concomitant statin use [34, 40]. Local delivery may be preferable for anterior segment disease or retinal indications requiring higher intraocular exposure with lower systemic risk. In DCN, topical fenofibrate produced earlier improvement than oral therapy and enhanced tear neurotrophic signaling [48], suggesting that targeted ocular delivery may confer faster or more specific benefits. Emerging approaches, including nano-emulsions, nanoparticles, hydrogels, sustained-release models, and microemulsions may improve ocular residence time, tissue targeting, and dosing durability [51, 95, 96, 117, 118, 128]. However, these local delivery strategies remain largely experimental, and further work on ocular pharmacokinetics, tissue penetration, formulation stability, long-term safety, and practical manufacturability is required before they can be translated into reliable clinical therapies.

Alongside questions of route and formulation, the clinical applicability of systemic fenofibrate must be weighed against its safety profile, particularly if long-term treatment is envisaged as a disease-modifying strategy [119]. Although fenofibrate is widely used in general medicine, ophthalmic repurposing would require structured baseline and periodic monitoring.

Renal safety is a consideration. Modest, reversible increases in serum creatinine are described [146], and dose reduction is recommended in renal impairment [34, 40]. Concomitant use with nephrotoxic agents, particularly cyclosporine, has been associated with an increased risk of nephrotoxicity, myositis, and rhabdomyolysis, and therefore warrants particular caution [34, 40]. Notably, despite these creatinine changes, long-term trials have shown a slower decline in eGFR and reduced progression of albuminuria compared with placebo, suggesting a net renoprotective effect [146].

Hepatic and muscle safety also require attention. Fenofibrate is contraindicated in active liver disease or unexplained persistent liver function test abnormalities, and routine monitoring of liver enzymes is advised [34, 40]. Skeletal muscle adverse effects range from myalgia and moderate creatine kinase elevation to rare rhabdomyolysis [34], although no rhabdomyolysis cases were reported in ACCORD or FIELD [40]. The risks of liver enzyme elevation and myopathy appear higher than with statin monotherapy [147], but fenofibrate is considered safer than gemfibrozil in combination with statins because it does not inhibit hepatic statin glucuronidation [148].

## Limitations of current evidence

Several limitations of the current evidence should be acknowledged. The strength of evidence across indications remains uneven: FIELD, ACCORD Eye, and LENS provide strong clinical support for fenofibrate in DR, but most other ocular indications rely largely on preclinical data. Even within DR and DME, many studies are secondary analyses, subgroup analyses, retrospective cohorts, or small interventional trials rather than large randomized trials, increasing the risk of residual confounding, outcome misclassification, and overinterpretation [120–123, 125]. Preliminary clinical studies in DCN is similarly limited by small sample sizes, open-label designs, lack of randomized placebo-controlled diabetic comparator groups, short treatment duration, and recruitment from single-country specialist centers, thereby limiting both causal inference and generalizability [35, 74].

Trial-level limitations also temper interpretation. ACCORD Eye had incomplete follow-up and limited retinal imaging time points, while ACCORDION suggested that DR benefit may not persist after treatment cessation, although this extension was limited by low retention and post-trial convergence of glycemic and lipid differences [42, 119]. LENS strengthened the evidence base but used screening-based retinal grading, single-field photography, and did not routinely include OCT, which may have reduced sensitivity for subtle DR or DME changes [46]. Available DME trials remain small and short, and are inconsistent in demonstrating visual acuity benefit despite some anatomical improvements [126, 127]. Interpretation is further complicated by heterogeneity in baseline disease severity and non-equivalent endpoints, including incident macular edema, treatment-requiring DME, OCT-based retinal thickness, and visual acuity outcomes [46, 126, 127].

Preclinical and mechanistic studies provide important biological plausibility but also have significant limitations. Many animal studies use young, male, single-strain models with short treatment windows, while some employ preventive rather than therapeutic protocols, which may not accurately reflect the complexity, chronicity, and treatment timing encountered in real-world human disease [31, 37, 38, 57, 91–93, 95, 117]. *In vitro* studies often rely on immortalized or simplified cell systems exposed to isolated stressors such as high glucose, IL-1β, 4-HNE, oxidized/glycated LDL, or hydrogen peroxide [24, 38, 39, 110–113, 115, 117]. These models are useful for pathway discovery but only partially reproduce chronic, heterogeneous human ocular disease. As noted earlier, tissue distribution, dose-response relationships, optimal treatment timing, and long-term ocular safety still remain insufficiently defined for novel ocular drug delivery systems [51, 95, 96, 117, 118, 128].

## Future directions

Future research should clarify where fenofibrate fits within ophthalmic disease management and which patients derive the greatest benefit. In DR and DME, adequately powered trials must determine whether fenofibrate primarily functions as an early progression-modifying therapy, to distinguish prevention of DME progression from treatment of established center-involving edema, and to assess whether adjunctive use reduces the cumulative burden of invasive retinal interventions. Future studies should separate prevention from treatment and stratify patients by disease severity, and concomitant therapies. Longer-term studies incorporating frequent, longitudinal retinal imaging are also needed to evaluate the durability of benefit after treatment discontinuation and characterize the trajectory of DR progression after drug cessation.

Larger controlled trials are similarly needed for DCN and other corneal and retinal diseases, where current evidence remains preliminary or predominantly preclinical. In DCN, future trials should compare fenofibrate against current symptomatic treatments (e.g., topical lubricants, tear substitutes, and nerve-targeted therapies) to confirm whether fenofibrate produces a meaningfully greater benefit than symptom management alone. Mechanistic studies should further define the relative contributions of PPARα-dependent and independent pathways, particularly the interplay among mitochondrial dysfunction, oxidative stress, cGAS-STING signaling, NF-κB activation, angiogenesis, and fibrosis. Finally, for newer ocular delivery systems, ocular pharmacokinetics, long-term safety, and optimal treatment strategies remain to be established across ophthalmic indications.

## Conclusion

**FIGURE 1:**
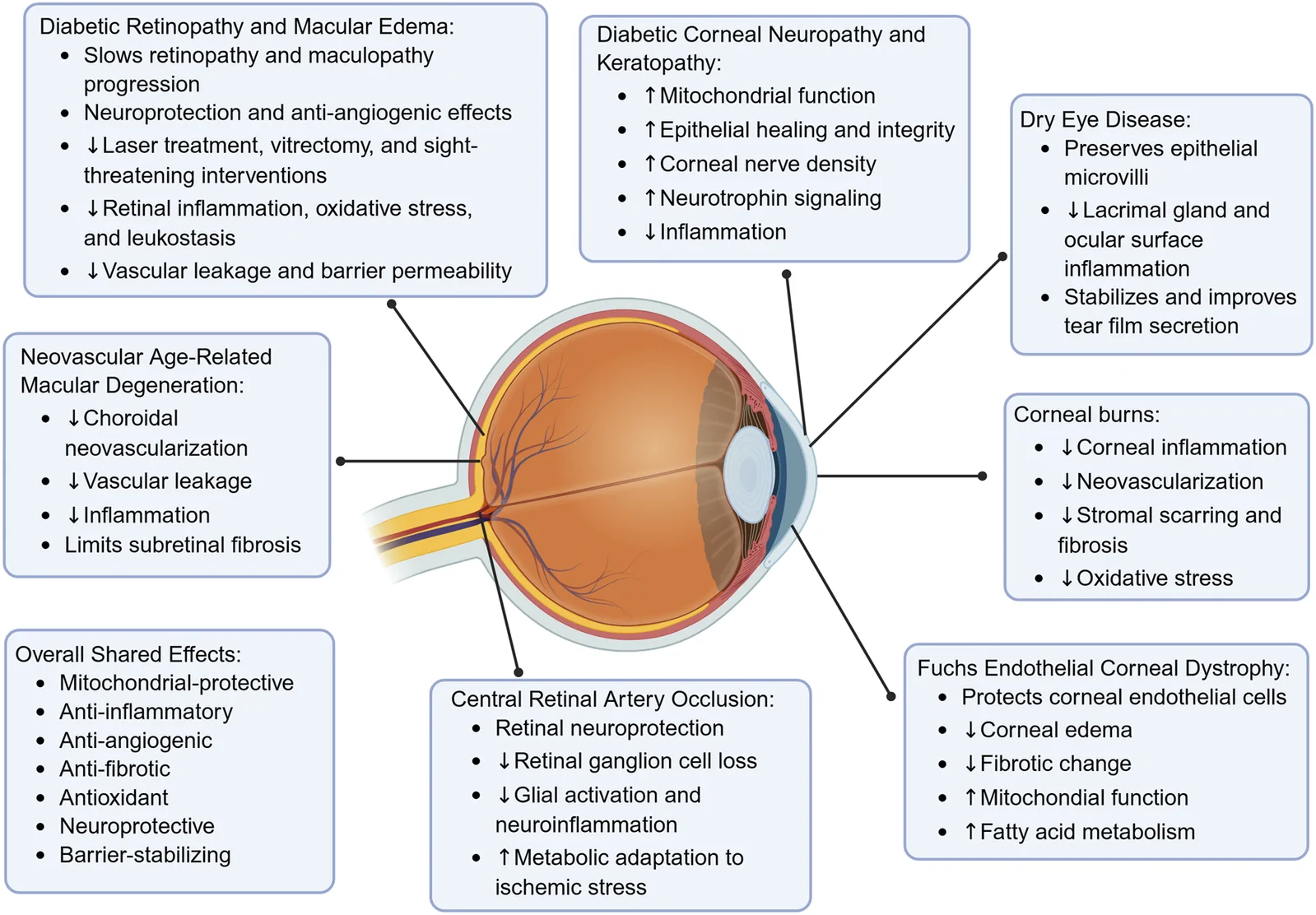
Illustration of the therapeutic effects of fenofibrate across corneal and retinal diseases. Created in BioRender. Liu, Y. C. (https://BioRender.com/60dqd3c) is licensed under CC BY 4.0.

In conclusion, fenofibrate shows promising potential across multiple ophthalmic diseases through effects on mitochondrial function, vascular stability, and tissue remodeling, with experimental data using its active metabolite FA further suggesting stabilizing actions on the blood-retinal barrier. (Figure 1). Current evidence most strongly supports its adjunctive role in slowing DR progression and reducing the need for retinal interventions in patients with mild-to-moderate DR, whereas its role in DME remains less certain and appears more preventive than therapeutic for established edema or visual acuity improvement. Emerging early clinical evidence also suggests potential benefit in DCN, particularly for corneal epithelial integrity, ocular surface inflammation, and corneal nerve regeneration. For DED, corneal burns, FECD, nAMD, and CRAO, evidence remains largely preclinical and is currently insufficient to establish clinical efficacy. Future research should prioritize larger controlled trials, validation of proposed mechanisms, optimized ocular drug-delivery strategies, and long-term safety evaluation to more clearly define fenofibrate’s role in ophthalmic practice. These findings may guide the development of more targeted, potent, and tissue-specific therapies. In this sense, fenofibrate may also serve as a stepping stone for broader therapeutic advances across corneal and retinal disease.
